# Psychometric Properties of The Persian Version of The Prenatal Attachment Inventory in Pregnant Iranian Women 

**DOI:** 10.22074/ijfs.2016.4908

**Published:** 2016-06-01

**Authors:** Reza Omani Samani, Saman Maroufizadeh, Zahra Ezabadi, Leila Alizadeh, Samira Vesali

**Affiliations:** Department of Epidemiology and Reproductive Health, Reproductive Epidemiology Research Center, Royan Institute for Reproductive Biomedicine, ACECR, Tehran, Iran

**Keywords:** Attachment, Pregnancy, Infertility

## Abstract

**Background:**

In 1993, Muller developed the Prenatal Attachment Inventory (PAI) which has been used widely in many studies and translated into several languages. The current study aimed to translate the PAI into Persian, assess the underlying structure of the PAI, and the appropriateness of the one-factor solution proposed by Muller.

**Materials and Methods:**

In this cross-sectional study, we recruited a total of 322 primi- gravidae in their 27^th^ to 34^th^ gestational weeks that referred to private and governmental prenatal clinics in Tehran, Iran. All participants completed the Persian versions of the PAI and a demographic questionnaire. Participants were re-tested 2 weeks after the initial test- ing. The following psychometric properties of the PAI were investigated: construct validity by confirmatory factor analysis (CFA), internal consistency reliability with Cronbach’s alpha, and test-retest reliability according to the intraclass correlation coefficient (ICC).

**Results:**

The CFA results indicated that a single-factor model provided good fit to the data, which confirmed the original model by its developer. The Cronbach’s alpha coef- ficient for PAI was 0.856 and the test-retest reliability with ICC was 0.784. Consider- ing the duration between marriage and pregnancy, women with low duration scored significantly higher than women with high duration on PAI (P=0.043).

**Conclusion:**

The Persian version of the PAI showed that one factor structure was ad- equate and could be used for measuring psychological affectionate attachment between Iranian mothers and their fetuses.

## Introduction

In 1981, Cranley initially definedmaternal-fetal attachment (MFA) as the extent to which “women engage in behaviors that represent an affiliation and interaction with their unborn child” (1). Then, Muller (2) presented another definition: “the unique and affectionate relationship that develops between a woman and her fetus”. Muller developed the Prenatal Attachment Inventory (PAI) (3) which has been continuously used as an instrument to measure psychological affectionate attachment between a mother and her fetus (4). 

It is believed that the relationship between a mother and herchild originates during pregnancy (5,8). Numerous conditions may affect the psychological status of a pregnant woman, resulting in change to the feto-maternal attachment. For example, there are reports that twin pregnancy, a history of infertility or infertility treatment, high risk pregnancy (9), maternal age (10), maternal mood (11,14), awareness of the fetus status by ultrasound (15), socio-economic levels (16), adequate prenatal care (17), pre-implantation genetic diagnosis (18), diet (19), a history of abortion (20), and exercise (21) affect feto-maternal attachment. Attachment can be an indicator for certain preand post-natal psychological disorders in mothers (22). 

PAI has been used in many prenatal studies worldwide in different languages and cultures (23,29). Each questionnaire and inventory must be adjusted with the country of the study, especially in terms of attitudes, beliefs, and emotions. Prior to research on the Iranian population, this inventory must be translated into Persian and evaluated prior to its use for research in Iran. Other studies have assessed and reported the PAI as single factor, three-factor, or five-factor structures. In a study by Pallant et al. (30), confirmatory factor analysis (CFA) of the original 21-item version of the PAI revealed poor fit to the model. These researchers supported a three-factor structure. The aim of the current study was to translate the PAI into Persian, primarily assess the underlying structure of the PAI, and the appropriateness of the one-factor solution previously proposed. 

## Materials and Methods

We used the forward-backward method to translate the PAI into Persian. The original inventory (3) was first translated from English to Persian, then from Persian to English, and again from English to Persian. Each translation was performed by a separate independent health staff member proficient in the English language. Cultural changes were as follows. In the 5th question: “I let other people put their hands on my tummy to feel the baby move”. In Islamic contexts, another person is not permitted to touch a woman’s body except her intimates “maharem”. Therefore, we have changed the question to: “I let my intimates put their hands on my tummy to feel the baby’s movement”. In the 8th question: “I tell others what the baby does inside me”. In Iran, most females are modest and shy, particularly with regards to issues related to reproduction and sexuality. They normally do not discuss these issues with others, especially those who reside in smaller towns and villages. We have changed this item to: “I tell my friends and relatives what the baby does inside of me”. 

### Content validity

After adjusting the questionnaire according to cultural, social, and religious ideas to prevent any bias from opposing beliefs, a group of sociologists, gynecologist, psychologist, clergies, and law experts carefully reviewed the questionnaire and exchanged their ideas in a group meeting. All group members were well familiar with reproductive health. 

### Face validity

After the final editing and best design of the questionnaire, we distributed it among 22 firsttime pregnant women in the 27 ^th^to 34 ^th^gestational week of pregnancy. An expert midwife with adequate education to avoid bias conducted the questionnaire via one-on-one interviews. After reviewing the results of the interview, we develop another edition and corrected the structure of the questionnaire according to the Persian language. 

### Prenatal attachment inventory

The PAI is a self-reporting instrument that consists of 21 items. Each item is scored on a 4-point Likert scale where 1=almost never, 2=sometimes, 3=often, and 4=almost always. Examples of items The PAI is a self-reporting instrument that consists of 21 items. Each item is scored on a 4-point Likert scale where 1=almost never, 2=sometimes, 3=often, and 4=almost always. Examples of items on the scale include: ‘‘I wonder what the baby looks like’’, ‘‘I know when my baby is asleep’’, and ‘‘I try to imagine what the baby is up to.’’ Total scores can range from 21 to 84, with higher scores indicative of higher levels of prenatal attachment. 

### Demographic characteristics

The demographic information questionnaire included age, duration from marriage to pregnancy, education level, occupation, and type of pregnancy (wanted or unwanted). 

### Participants

In this cross-sectional study, we assessed the reliability of the PAI by administering this questionnaire to 322 first-time pregnant women in their 27 ^th^to 32 ^nd^gestational weeks. The women referred to private and governmental prenatal clinics in Tehran, Iran. The questionnaire was administered to these women again after 10-12 days. Inclusion criteria were: being able to read and write Persian, over 18 years of age, low-risk pregnancy, gestational age of over 25 weeks, and no previous abortions. We excluded women younger than 18 years of age because they presumably have experienced stress which could influence maternal attachment. We also excluded high-risk pregnancy and abortion because these events might lead to a different type of attachment to the fetus. 

### Ethical consideration

The Ethics Committee at Royan Institute approved this study. All participants received information about the purpose of this study and gave their verbal informed consent to participate. 

### Statistical analysis

CFA was used to examine the factor structure of the PAI. The fit indices we have employed to test the model fit included: chi-square (χ2), relative chi-square [χ2/degree of freedom (df)], comparative fit index (CFI), root mean square error of approximation (RMSEA), and the standardized root mean square residual (SRMR). A non-significant χ2 statistic indicates a good model fit (P>0.05). Unfortunately, the χ2 statistic is highly sensitive to sample size, especially if the observations are greater than 200. An alternate evaluation of the χ2 statistic is to examine the χ2/df for the model. A χ2/df ratio of 3 or less is indicative of a good model fit. Values of CFI>0.9, SRMR<0.08, and RMSEA<0.08 indicate good fit with the data. Internal consistency of the PAI was examined using Cronbach’s alpha coefficient and test-retest reliability of the scale by ICC. 

All statistical analyses were performed using SPSS version 16.0 (SPSS Inc., Chicago, IL, USA), except for the CFA, which was performed using Lisrel 8.80 (Scientific Software International, Inc., Lincolnwood, IL, USA). All statistical tests were two-tailed and a P value<0.05 was considered statistically significant. 

## Results

### Participants’ characteristics

Table 1 lists the socio-demographic characteristics of the participants. Participants had a mean age of 28.57 ± 4.13 years (range: 18 to 43 years). Of participants, the majority were housewives (63.8%), 54.4% had college or university degrees, and 93.1% wanted to become pregnant. The mean duration from marriage to pregnancy was 4.31 ± 2.75 years. 

**Table 1:** Socio-demographic characteristics of the participants

Age (Y)	28.57 ± 4.13
Duration from marriage to pregnancy (Y)	4.31 ± 2.75
Education level	n (%)
Elementary	14 (6.0)
Secondary	92 (39.6)
University	126 (54.4)
Occupation	
Employed	79 (34.1)
Housewife	148 (63.8)
Student	5 (2.1)
Type of pregnancy	
Wanted	216 (93.1)
Unwanted	16 (6.9)

### Reliability analysis

Cronbach’s alpha coefficient for assessing in- ternal consistency of the PAI was 0.856. The 2-week test-retest reliability with ICC was 0.784.

### Confirmatory factor analysis

The CFA was performed to determine the fit of the previously identified one-factor model. The goodness of fit indices revealed that the single-factor model was a good fit to the data (χ2=532.36, df=189, P<0.001, χ2/df=2.82, CFI=0.90, RMSEA=0.089, and SRMR=0.078). All standardized factor loadings were signifi- cant, in the expected direction, and ranged from 0.29 to 0.64 (data not shown).

### Comparison of the Prenatal Attachment Inven- tory by type of pregnancy and duration from marriage to pregnancy

We used the independent samples t test to examine the differences between PAI, type of pregnancy, and duration from marriage to preg- nancy. There was no significant difference between groups of wanted pregnancies and unwanted pregnancies on the PAI (P=0.945). The results indicated that women with low duration (64.14 ± 9.12) scored significantly higher than women with high duration (61.68 ± 9.24) between marriage and pregnancy on the PAI (P=0.043) (Table 2). 

**Table 2:** Comparison of the Prenatal Attachment Inventory (PAI) by type of pregnancy and duration from marriage to pregnancy


	n	Mean (SD)	t	P value

Type of pregnancy			0.07	0.945
Wanted	216	62.83 (9.20)		
Unwanted	16	63.00 (10.15)		
Duration from marriage to pregnancy			2.03	0.043
<4 years	110	64.14 (9.12)		
≥4 years	122	61.68 (9.24)		


## Discussion

This is the first study to assess psychometric properties of the PAI in pregnant Iranian women. PAI is a well-known questionnaire for measurement of feto-maternal attachment. This questionnaire has been translated into several languages and used in numerous countries (24-29). The PAI has been used to produce new questionnaires (31-36). Culture and beliefs of a society may impact attachment between a mother and her infant (31), and attitude towards the unborn child is different in various parts of the world. Therefore, it is important to conduct research in order to prove any relation between demographic variables, education, and socioeconomic levels to prenatal attachment (9, 10, 32).

The current study demonstrated that the one-factor structure of the questionnaire had adequate psychometric properties. CFA results showed that the one-factor structure of the PAI had good psychometric properties with adequate internal consistency. Pallant et al. (30) reported that the CFA of a single-factor was a poor fit to the model and the three-factor solution was the most appropriate to represent the PAI items. Cronbach’s alpha above 0.70 showed appropriate internal consistency among the questions so that it could be used in the Iranian context as a good inventory to measure attachment between a mother and her fetus. It would explain the psychological connection between a pregnant woman and her unborn child. Another study has reported a variation in the behavior of the individual PAI subscales during both the prenatal and postnatal periods. The reliability of the total PAI scale reported was acceptable (Cronbach alpha=0.86) (36). In this study confirmed the external validity of tool by test-retest reliability. An ICC equal to 0.784 showed a very good correlation in repeating the test during the time interval. Pallant et al. (30) demonstrated that the three-factor inventory had adequate internal consistency and reliability (above 0.7).

The results of the independent samples t-test showed a significantly high prenatal attachment relationship in women who had a slight time difference between their marriage and pregnancy.

As mentioned before, prenatal attachment may predict future relations between a mother and her child (30). Thus, it would be of benefit to determine factors that affect this relationship and discover methods to decrease prenatal attachment reducing factors to help the future of a mother and child. It has also been reported that factors such as genetic screening (37-39), twin pregnancy (23, 40), trauma (41), maternal age (10), maternal mood (11-14), and miscarriage (20) affect the MFA. There may be a correlation between prenatal and postnatal attachment (24). A growing number of studies report the impact of prenatal attachment on subsequent postnatal bonding (36), however further studies are necessary to better understand its effect on the mother’s adjustment to the parenting role, the mother-child relationship, and the development and well-being of the child. There should be additional studies that pertain to influencing factors in different parts of the world, particularly Middle Eastern countries. 

The Persian version of the PAI showed that one factor structure is adequate and can be used for measuring psychological affectionate attachment between Iranian mothers and their fetuses.

## Conclusion

The Persian version of the PAI showed that one factor structure is adequate and can be used for measuring psychological affectionate attachment between Iranian mothers and their fetuses.

## References

[1] Cranley MS (1981). Development of a tool for the measurement of maternal attachment during pregnancy. Nurs Res.

[2] Muller ME (1993). Development of the prenatal attachment inventory. West J Nurs Res.

[3] Müller ME (1996). Prenatal and postnatal attachment: a modest correlation. J Obstet Gynecol Neonatal Nurs.

[4] McMahon CA, Ungerer JA, Beaurepaire J, Tennant C, Saunders D (1997). Anxiety during pregnancy and fetal attachment after in-vitro fertilization conception. Hum Reprod.

[5] Gaffney KF (1988). Prenatal maternal attachment. Image J Nurs Sch.

[6] Bialoskurski M, Cox CL, Hayes JA (1999). The nature of attachment in a neonatal intensive care unit. J Perinat Neonatal Nurs.

[7] Cranley MS (1981). Roots of attachment: the relationship of parents with their unborn. Birth Defects Orig Artic Ser.

[8] Grace JT (1989). Development of maternal-fetal attachment during pregnancy. Nurs Res.

[9] Kemp VH, Page CK (1987). Maternal prenatal attachment in normal and high-risk pregnancies. J Obstet Gynecol Neonatal Nurs.

[10] Lerum CW, LoBiondo-Wood G (1989). The relationship of maternal age, quickening, and physical symptoms of pregnancy to the development of maternal-fetal attachment. Birth.

[11] Honjo S, Arai S, Kaneko H, Ujiie T, Murase S, Sechiyama H (2003). Antenatal depression and maternal-fetal attachment. Psychopathology.

[12] Lindgren K (2001). Relationships among maternal-fetal attachment, prenatal depression, and health practices in pregnancy. Res Nurs Health.

[13] Hart R, McMahon CA (2006). Mood state and psychological adjustment to pregnancy. Arch Womens Ment Health.

[14] Seimyr L, Sjogren B, Welles-Nystrom B, Nissen E (2009). Antenatal maternal depressive mood and parental-fetal attachment at the end of pregnancy. Arch Womens Ment Health.

[15] Boukydis CF, Boukydis CF, Treadwell MC, Delaney-Black V, Boyes K, King M (2006). Women's responses to ultrasound examinations during routine screens in an obstetric clinic. J Ultrasound Med.

[16] Walker LO, Cooney AT, Riggs MW (1999). Psychosocial and demographic factors related to health behaviors in the 1st trimester. J Obstet Gynecol Neonatal Nurs.

[17] Lowry LW, Beikirch P (1998). Effect of comprehensive care on pregnancy outcomes. Appl Nurs Res.

[18] Karatas JC, Barlow-Stewart K, Meiser B, McMahon C, Strong KA, Hill W (2011). A prospective study assessing anxiety, depression and maternal-fetal attachment in women using PGD. Hum Reprod.

[19] Abrams B, Altman SL, Pickett KE (2000). Pregnancy weight gain: still controversial. Am J Clin Nutr.

[20] Tsartsara E, Johnson MP (2006). The impact of miscarriage on women's pregnancy-specific anxiety and feelings of prenatal maternal-fetal attachment during the course of a subsequent pregnancy: an exploratory follow-up study. J Psychosom Obstet Gynaecol.

[21] Clapp JF (2000). Exercise during pregnancy.A clinical update. Clin Sports Med.

[22] Monk C, Leight KL, Fang Y (2008). The relationship between women's attachment style and perinatal mood disturbance: implications for screening and treatment. Arch Womens Ment Health.

[23] Damato EG (2000). Maternal-fetal attachment in twin pregnancies. J Obstet Gynecol Neonatal Nurs.

[24] Damato EG (2004). Prenatal attachment and other correlates of postnatal maternal attachment to twins. Adv Neonatal Care.

[25] Hjelmstedt A, Widstrom AM, Collins A (2006). Psychological correlates of prenatal attachment in women who conceived after in vitro fertilization and women who conceived naturally. Birth.

[26] Stanton F, Golombok S (1993). Maternal-fetal attachment during pregnancy following in vitro fertilization. J Psychosom Obstet Gynaecol.

[27] Armstrong D, Hutti M (1998). Pregnancy after perinatal loss: the relationship between anxiety and prenatal attachment. J Obstet Gynecol Neonatal Nurs.

[28] Lawson KL, Turriff-Jonasson SI (2006). Maternal serum screening and psychosocial attachment to pregnancy. J Psychosom Res.

[29] Siddiqui A, Hagglof B (2000). Does maternal prenatal attachment predict postnatal mother-infant interaction?. Early Hum Dev.

[30] Pallant JF, Haines HM, Hildingsson I, Cross M, Rubertsson C (2014). Psychometric evaluation and refinement of the prenatal attachment Inventory. J Reprod Infant Psychol.

[31] Mercer RT (1986). Predictors of maternal role attainment at one year postbirth. West J Nurs Res.

[32] Chen CJ, Chen YC, Sung HC, Kuo PC, Wang CH (2011). Perinatal attachment in naturally pregnant and infertility-treated pregnant women in Taiwan. J Adv Nurs.

[33] Huang HC, Wang SY, Chen CH (2004). Body image, maternalfetal attachment, and choice of infant feeding method: a study in Taiwan. Birth.

[34] Shieh C, Kravitz M (2002). Maternal-fetal attachment in pregnant women who use illicit drugs. J Obstet Gynecol Neonatal Nurs.

[35] Jurgens MA, Levy-Rueff M, Goffinet F, Golse B, Beauquier-Macotta B (2010). [Psychometric properties of the French version of the prenatal attachment inventory in 112 pregnant women]. Encephale.

[36] Bielawska-Batorowicz E, Siddiqui A (2008). A study of prenatal attachment with Swedish and Polish expectant mothers. J Reprod Infant Psychol.

[37] Kleinveld JH, Timmermans DR, van den Berg M, van Eijk JT, Ten Kate LP (2007). Does offering and performing prenatal screening influence women's attachment to their unborn child?. A longitudinal randomized controlled trial. Prenat Diagn.

[38] Gau ML, Lee TY (2003). Construct validity of the prenatal attachment inventory: a confirmatory factor analysis approach. J Nurs Res.

[39] Rowe H, Fisher J, Quinlivan J (2009). Women who are well informed about prenatal genetic screening delay emotional attachment to their fetus. J Psychosom Obstet Gynaecol.

[40] Brandon AR, Pitts S, Robinson R, Stringer CA (2007). Maternal and fetal representations, dimensions of personality, and prenatal attachment in women hospitalized with high-risk pregnancy. J Am Psychoanal Assoc.

[41] Schwerdtfeger KL, Goff BS (2007). Intergenerational transmission of trauma: exploring mother-infant prenatal attachment. J Trauma Stress.

